# Identifying fear of childbirth in a UK population: qualitative examination of the clarity and acceptability of existing measurement tools in a small UK sample

**DOI:** 10.1186/s12884-020-03249-4

**Published:** 2020-09-22

**Authors:** P. Slade, K. Balling, K. Sheen, G. Houghton

**Affiliations:** 1 Department of Primary Care and Mental Health, Institute of Population Health, University of Liverpool, Ground Floor Whelan Building, Brownlow Hill, Liverpool, L69 3GB UK; 2 Natural Sciences and Psychology, Liverpool John Moores University, Liverpool, UK; 3 Liverpool Women’s Hospital Foundation Trust, Crown Street, Liverpool, L8 7SS UK

**Keywords:** Antenatal anxiety, Anxiety measure, Childbirth anxiety, Fear of childbirth, Tokophobia

## Abstract

**Background:**

Fear of childbirth is related to but not synonymous with general anxiety, and represents a superior predictor for maternal and infant outcomes. There is a need to improve the identification and provision of support for women experiencing high fear of childbirth. However it is uncertain as to whether existing measurement tools have appropriate content validity (i.e. cover the relevant domains within the construct), practical utility, and whether they are acceptable for use with a UK population. This study aimed to (1) identify the utility and acceptability of existing measures of fear of childbirth (FOC) with a small UK sample and (2) map the content of existing measures to the key concepts of fear of childbirth established by previous research.

**Methods:**

Ten pregnant women; five with high and five with low fear of childbirth participated in a cognitive interview covering four most commonly used measures of fear of childbirth: 1. The Wijma Delivery Expectancy Questionnaire (WDEQ A), 2. The Oxford Worries about Labour Scale (OWLS), 3. The Slade-Pais Expectations of Childbirth Scale – fear subscale (SPECS) and 4. The Fear of Birth scale (FOBS). Each measure was also reviewed by participants for ease and clarity of understanding and acceptability. The measures were then reviewed against the key domains identified in the fear of childbirth literature to ascertain the adequacy of content validity of each measure. Interviews were analysed using thematic analysis for each scale item.

**Results:**

All measures except the FOBS, included items that either women did not understand or, if where there was understanding the meanings were inconsistent across women. All measures demonstrated limited acceptability and content validity for the specific construct of FOC. Therefore, none of the measurement tools currently used within the UK met criteria for understanding, acceptability and content validity for measurement of FOC.

**Conclusions:**

Findings emphasise a need to develop a specific fear of childbirth tool with good clarity which demonstrates appropriate content validity, and that is acceptable in presentation and length for pregnant women in a UK population.

## Background

Fear of childbirth can have adverse impacts on women’s experience of birth and birthing outcomes [1–3]. Research on prevalence rates for FOC show extensive worldwide variation, ranging from 1.9 to 30% [4–6]. This may be due to genuine cultural differences, or a reflection of the lack of clarity in the definition of the term FOC alongside (or in conjunction with) the diversity and adequacy of measurement tools [6, 7].

General measures of anxiety have often been used as proxy measures for FOC. However, although there is an element of comorbidity with general anxiety, the two responses are not synonymous [8]. The level of fear and anxiety specific to pregnancy and birth is a superior predictor for maternal and infant outcome over general anxiety alone [9]. This emphasises the need for specific assessment of FOC. Although specific assessment tools for FOC do exist, their utility for use with a UK population is currently unclear.

Fear relating to birth occurs on a spectrum, and when at its most severe it can resemble a phobic response [10]. Interpretation of the severity of FOC has been limited by variations in the thresholds applied to scores from self-report questionnaires, and in the timing of measurement during pregnancy [11, 12]. This is particularly pertinent for a UK sample where translations without validation have been used. Timing is a pertinent issue for the measurement of FOC, and has received limited systematic investigation to date. In addition, the acceptability and clinical utility of existing measurement tools for a UK population has not yet been evaluated.

Underpinning these problems was the absence of a clear definition for FOC [12]. However a recent investigation has systematically explored the experiences of FOC and has identified 10 domains [13]. These now provide a basis upon which to examine whether existing measures provide comprehensive assessment of the elements that women fear about birth.

### Current measures for fear of childbirth

A literature search was conducted by members of the research team (KS, PS) to identify current measures used to assess fear or concerns relating to pregnancy and childbirth using WOK, SCOPUS and EBSCO (including Medline and CINAHL) databases. Search terms included “anxiety”, “fear” and “preg”, “antenatal”, “birth”. Empirical studies assessing fear, anxiety, concerns or worries about pregnancy and/or childbirth were identified and their method of assessment extracted. Review papers were included and additional references hand searched. Studies involving assessment of fear/anxiety/stress specific to childbirth with participants who were either pregnant and/or postpartum were included. There was no exclusion on the basis of parity of the sample. Exclusion criteria were: not assessing psychological anxiety or fear relating to childbirth, not assessing psychological appraisal but the availability of tangible assets (e.g. financial information), not a childbearing sample.

Scales that met the inclusion criteria, where item content focussed on fear or concerns specific to birth, were selected for further evaluation. Four main scales were identified: Wijma Delivery Expectancy Questionnaire Version A (WDEQ-A) [14], Fear of Birth Scale (FOBS) [15], the Oxford Worries about Labour Scale (OWLS) [16], and the Slade-Pais Expectations of Childbirth Scale (SPECS Fear subscale) [17].

The Wijma Delivery Expectancy Questionnaire Version A (WDEQ-A) assesses expectancies of childbirth [14].Version B (WDEQ-B) measures experience and is used postnatally so is not relevant here. The 33 item measure is the most frequently used questionnaire to measure FOC [12]. Responses on WDEQ-A are recorded on a scale of 0 (extremely) to 5 (not at all); total scores range from 0 to 165, and threshold scores for high (> = 66) or severe (> = 86) FOC have been suggested [18, 19]. The scale has demonstrated good reliability and validity particularly in research settings [20–22]. The questionnaire has good internal consistency reliability and split-half reliability of greater than or equal to 0.87 [14]. Scores for nulliparous women have been identified as higher than for multiparous women using this scale [20, 23].

Although the WDEQ-A was developed in and has been extensively used in Sweden [14, 18, 19, 24, 25]. It has also been used in Norway [1, 8, 26], Australia [20, 27] and England [23]. Items were developed purely on the basis of clinical experiences of two of the authors [14]. Ensuring appropriate content validity for any questionnaire normally requires exploration of the relevant domains within the construct the scale aims to measure, and using the experiences of women themselves is recommended [28]. Furthermore studies using the WDEQ-A have identified major issues in item interpretation following translation into English [23, 27, 29].

The utility of existing cut-off scores for high FOC in UK populations have also been questioned. A recent systematic review highlighted significant heterogeneity in the application of threshold scores inferring FOC severity [12]. Zar, Wijma and Wijma [18] initially developed the threshold for low, moderate and high fear using the distribution of scores across each quartile in a study of women in Sweden (*n* = 196). The threshold for severe FOC was devised by Ryding et al. [19], by selecting scores in the upper tenth percentile in a study of women (*n* = 1981) in Sweden. Inference of severity using statistical distribution of scores that (1) may be specific to that cultural population and (2) has not been assessed in terms of implications for level of impairment may not be appropriate. Identification of thresholds associated with impairment to daily life and specific to the relevant population and/or culture would enable accurate identification of women with significant FOC who may benefit from intervention. The clinical utility of cut off scores on the WDEQ-A was recently examined in Italy [11], however validation with a UK population is yet to be completed. Studies with UK populations have reported higher mean scores for fear using the W-DEQ-A [23], which suggests that inference of severity based on normative scores from Swedish populations may be misleading.

The *Fear of Birth Scale* [15] (FOBS) is a measure which is increasingly gaining interest as a simple clinical tool to identify elevated levels of FOC. The FOBS assesses the extent to which women are experiencing fear and worry in relation to the approaching birth using two items recorded using a visual analogue scale (VAS). Using a 100 mm VAS scale women indicate the extent to which they have felt (1) calm/worried or (2) no fear/fear in relation to birth. Internal consistency of the scale is very good (α = .84), and the FOBS demonstrates a moderate correlation (r = .66, *p* = <.001) to total scores on the WDEQ-A [27]. Furthermore, an average score of 54 across both items has been found to effectively identify those reporting severe FOC on the WDEQ-A, with a sensitivity of 89% and specificity of 79% in a sample of Australian women [27].

The FOBS was generated using semi-structured interviews with a think aloud technique with 31 pregnant women (17–20 weeks gestation). Content analysis was then conducted to describe the different dimensions of fear of birth. Whilst the utility of the scale has been assessed against the WDEQ-A in a large Australian cohort [30], the clinical utility of this scale within the UK is uncertain and warrants further exploration. Use of the FOBS has recently been supported as a clinically effective way to open discussions around fear of childbirth, prior to in-depth exploration of fears if present [12, 31].

The *Oxford Worries about Labour Scale* [16](OWLS) assesses worries around labour and delivery. The 10-item measure was developed and psychometrically assessed with a large pilot sample of women in England (*n* = 240) reporting on women’s experiences of maternity care in England as part of a large survey (*n* = 2697). Responses are scored on a 4-point scale ranging from 1 (very worried) to 4 (not worried at all), and total scores range between 1 to 40. Exploratory factor analysis identified three sub-scales relating to fears about 1) labour pain and distress, 2) pre-labour uncertainty and 3) interventions. The scale has demonstrated good construct and divergent validity [16] but it has received limited use and requires evaluation for utility in prospective designs.

The *Slade-Pais Expectations of Childbirth Scale* [17] (SPECS) measures women’s expectations of childbirth. The full scale consists of 50 items and six factors, scored on a 5 point scale ranging from 1 (strongly agree) to 5 (strongly disagree). The fear subscale (10 items) specifically assesses fears about giving birth. Scores on this subscale range between 10 and 50. Validity and reliability within a UK sample has yet to be fully reviewed, however an initial indication of psychometric robustness suggests acceptable internal reliability and good construct validity [17]. Items were developed from semi-structured interviews with 18 pregnant women in England, where their expectations around childbirth were explored. Further testing with 148 pregnant women demonstrated promising psychometric properties. The dimensions of the SPECS reflect key areas highlighted in previous literature about the type of expectations held by women prior to giving birth, including levels of control, pain, fear, support from partners and healthcare staff and positive anticipations of giving birth. Further evaluation of the scale is required if used as a standalone measure for FOC [17].

Despite a number of measures available to assess FOC in the UK, it is uncertain whether these measures fulfil basic requirements of clarity and acceptability to pregnant women. Cognitive interviews are a well established methodology which enables the identification of any issues in clarity and acceptability across participants [32–34].More specifically, as indicated by Collins et al. [34] they can be used to check if ‘(i) respondents can understand the question concept or task,(ii) they do so in a consistent way, and,(iii) in a way the researcher intended’. These are clearly key issues in whether a questionnaire has utility in a particular cultural context. In addition to clarity and acceptability, scales must also demonstrate appropriate content validity in terms of the range of domains covered..

### Aim

1. To evaluate four current tools for FOC in terms of their adequacy for use with a UK population based on women’s understanding of the measure and the acceptability of completing the measure
2. To assess content validity of each measure against the 10 key elements of FOC [13].

## Methods

### Design and approach

To examine clarity and acceptability of the measurement tools the study used a qualitative research design employing cognitive interviewing. This is an approach used to identify their internal processes and opinions. It can be used as a tool to assess participants’ views on the clarity and acceptability of items whilst completing a measure. Cognitive interviewing is a method of establishing participants’ understanding of individual items [35]. It examines participants’ comprehension of each item, their ability to retrieve information required to provide an answer, make a judgement about the item’s relevance and provide a response that corresponds to the original purpose of the item. There are two techniques used in cognitive interviewing; think-aloud and verbal probing [35]. Think aloud techniques involve the researcher reading the items to the participant, who then verbalises their thoughts. Verbal probing involves additional specific questions that are asked to elicit further information. Both techniques were employed during the interviews. Rather than being informed via saturation, small sample sizes are sufficient to identify major issues in item clarity and acceptability and the recommended sample size for a cognitive interview design is 5 to 15 participants [32].

Content validity was examined via review against the 10 key elements of FOC [13].

### Participants

Pregnant women (*n* = 10) were recruited. As the primary objective of this element was to evaluate the comprehension and acceptability of items, purposive sampling with half women reporting low and half high levels of fear of childbirth was employed. Presence of elevated fear of childbirth was inferred via women’s self-report but also, for the majority, confirmed by the clinical judgement of the specialist midwife (GH).

Women aged 16 and over, fluent in spoken English in their second and third gestational phases stages of pregnancy were eligible to take part. Participants were excluded if they had a history of stillbirth or intrauterine death, had an ongoing serious maternal medical condition, if there was a medical concern for the baby in their current pregnancy, if they were under the care of the fetal medicine unit, or the perinatal mental health team or the enhanced midwifery team. Table 1 provides demographic characteristics.

**Table 1:** Demographics of participants

		Low fear (***n*** = 5)		High fear (***n*** = 5)	
		Range	Mean	Range	Mean
Age range		32–37	35	29–38	32
Gestation (weeks)		20–24	23		32
		**N**		**N**	
Marital Status	Single	0		1	
	Cohabiting/ married	5		4	
Previous children	Primiparous	2		2	
	Multiparous	3		3	

### Researcher characteristics

Women were made aware that the interviewer was a woman clinical psychologist with perinatal experience but no prior assumptions about how the measures to be covered would be appraised.

### Measures

The following measures were reviewed:

1. The Wijma Delivery Expectancy Questionnaire [14] (WDEQ-A)
2. The Fear of Birth scale [15] (FOBS)
3. The Oxford Worries about Labour Scale [16] (OWLS)
4. The Slade-Pais Expectations of Childbirth Scale, Fear subscale [17] (SPECS) –

### Procedure

Ethical approval was obtained from the University of Liverpool (15/NW/0922) and the study was sponsored by University of Liverpool (UoL00177).

Women who were currently experiencing FOC (*n* = 5) were recruited via a clinic run specifically for such difficulties by the consultant midwife (GH) at the Liverpool Women’s Hospital NHS Foundation Trust (LWHFT). Women who disclose FOC to their midwife during pregnancy are routinely referred to the clinic where they discuss their fears with the consultant specialist midwife. During the consultation, GH provided information about the study and asked them whether they would be willing to be contacted by the researcher (KB).

Women with low (or no) FOC (*n* = 5) were recruited primarily via a research midwife at their routine 20-week scan appointment. All participants were asked whether they would like to receive further information from the researcher.

Details of the study were also presented on the LWHFT website and LWHFT social media websites (Twitter, Facebook) to ensure that all pregnant women were given the opportunity to read about the study and were able to contact the researcher directly should they wish to. This was to enable those who may have difficulty disclosing fears to health professionals to be included.

The researcher (KB) then contacted women to provide further information about the aims and purposes of the study. In total 30 women requested further information; 12 women through self-referral having seen information via social media, 7 via referrals from the consultant midwife (for high fear) and 11 referrals from the research midwife (for low fear). After indicating willingness for contact, the main reason that women were not recruited was due to difficulty in actually making contact.

On receipt of written consent, the researcher met with the participant at a place of their preference where they participated in a cognitive interview, reviewing the four FOC measures (WDEQ-A, OWLS, SPECS, FOBS). Interviews with women were conducted at LWHFT (*n* = 2), in a woman’s home (*n* = 7) or at the University of Liverpool (*n* = 1). No repeat interviews were conducted.

All participants were introduced to the concept of cognitive interviewing and specifically the ‘thinking out loud’ by counting the windows in their home and verbalising their thoughts as they counted them. Each participant completed the measures in the same order. The interviews typically lasted around 60 min.

For each FOC measure the following criteria was reviewed. Elements one and two (understanding and acceptability) were reviewed via the cognitive interviews with women. Element three (content validity) was examined as a separate process.

1. Understanding - Whether the FOC measure was understood by women, and if so, whether the attributed meaning of the item was unambiguous i.e. all women reported a consistent understanding of the term. To further assess understandability, the reading ease for each measure was reviewed with the Flesch reading ease test, to identify the reading level needed to understand the measure. The test rates text on a 100 point scale (and the higher the score the easier it is to read). The public should aim for a Reading Ease score of around 60 or above, meaning that the text should be easily understood by a 13–15 year old.
2. Acceptability - whether the FOC measure is acceptable to women when completing the measure, in terms of its length, presentation and overall impression of the measure.
3. Exploration of content validity - whether the FOC measure has good content validity i.e. the items reflect the full range of domains of fear that women have of childbirth [36], as identified within a UK sample. To determine this, the dimension, each scale was reviewed by the research team (PS, GH, KB, KS) to identify items addressing each of the 10 key elements for fear of childbirth, as identified by Slade, Balling, Sheen & Houghton [13]. The elements include: 1) Fear of not knowing and not being able to plan for the unpredictable, (2) Fear of harm or stress to the baby, (3) Fear of their inability to cope with the pain, (4) Fear of their body’s ability to give birth, (5) Fear of harm to self in labour and postnatally (6) Fear of being ‘done’ to (7) Fear of not having a voice in decision making (8) Fear of being abandoned and alone (9) Fear of internal loss of control and (10) Terrified of birth and not knowing why.

### Data analysis

The interviews were audio recorded, transcribed ad verbatim, and analysed using thematic analysis [37] to identify (i) general understanding of each item in relation to clarity (this identified whether a single or multiple dimensions emerged within women’s responses). This then provided information on the consistency of understanding and (ii) women’s perspectives on the acceptability of each item. No field notes were made. NVivo was used to organise the data. Transcripts were not returned to participants for comment and participants did not provide feedback on the findings, although all participants were able to request an overview of findings.

Each stage of analysis was reviewed by the research team in terms of the documented evidence provided. Consensus on the adequacy of each item’s clarity of meaning and general acceptability as reported by participants was determined via a series of structured team discussions (PS,KB,KS,GH). Initially, the interview data were analysed in groups of women with low and high fear. There was a high degree of consensus among the two groups but differences, where they existed, are highlighted in the findings.

To examine content validity, all 4 measures were reviewed by the research team comprising of a consultant midwife (GH), consultant clinical psychologist (PS), clinical psychologist (KB) and research psychologist (KS).to explore whether items in each of the scale mapped across to each of the 10 key elements of fear of childbirth as identified by Slade et al. [13].

## Results

### Understanding and clarity of measures

#### WDEQ-a

Of the 33 items in the WDEQ-A, women found three items difficult to understand as follows: (1) ‘Frightful’, as many women with high fear exchanged the word ‘fearful’ or ‘scary’:

*“About a two … Just scared … It would be (frightful) for me but I don’t know whether it would be for the general public, for everybody. Yeah, I think fearful would be better than frightful.”*

(2) ‘Desolate’, the majority of women did not know what this word meant, and those that did, did not relate it to their pregnancy:

*“That word doesn’t mean anything me. I wouldn’t use it. To be honest I don’t even know what it means.”*

and (3) ‘As it should be’, many of the women did not understand what this question was trying to ask:

*“I don’t think I really kind of understand that either? I have to really think about what it means, that is not an easy question to answer, no.”*

For a further 7 items women lacked clear understanding due to ambiguity (i.e. the women attributed different meanings to the item) (1) ‘Strong’, although all women understood the meaning of the word strong, for the high fear group ‘strong’ could include being either strong mentally or strong physically:

*“so it’s like I’m not going to be strong, my body is not going to do what it’s supposed to do. So maybe yeah, maybe that is a word that I would use probably … Not mentally strong enough because I feel that I, you know, mentally you just have to adapt to the situation that you are in but physically there’s nothing you can do about your body is there?”*

Whereas for the low fear group, all women identified it as meaning mentally not physically strong:

*“It’s like a capacity to cope with it … Yeah, likes it’s an endurance test.”*

(2) ‘Weak’, similarly, to strong, women perceived weak to be either physically or emotionally weak, with no consensus:

*“Weak, not able to cope with it. That’s how I would look at it.”*

*“Weak is a physical one again. Yeah and that’s because I just think of weak as a physical not being able to tolerate it.”*

(3) ‘Independent’, this either was interpreted as being independent from other people i.e. nobody else going through the labour (independently facing the process):

*“Being able to cope on your own. Because obviously it is you that is going through it at the end of the day … ”*

or being independent minded to say what you want in labour:

*“So I felt this time, that I have been a little bit independent with speaking out and saying ‘well actually hang on you know I would like it this way, I would like it that way’ … I would say independent or in control.” Q2*

(4) ‘Self-confidence’, some felt that self-confidence didn’t relate to labour and birth and that it was more about how one perceives their appearance generally:

*“I suppose I could read it and understand what it means but I wouldn’t say to somebody “oh I felt really self-confident” I think I associate self-confidence more with like, you know the way you look or your personality rather than your ability or doing well in something.” Q10*

whereas others related it to their self-confidence to either get through the process or ask for help if they needed it during labour:

*“Self-confidence would probably be about how I feel within myself as a person and maybe I would relate it to more self-confidence to direct and have a say in the process.” Q30*

(5) ‘Lose control of myself’, this was perceived as a negative question and for some there was a lack of clarity about whether it refers to an emotional or physical loss of control:

*“Lose control like things are going to get so bad that it is a possibility that you would totally lose control of yourself. It’s a little bit scary that maybe that that’s a possibility” Q3*

(6) ‘Totally as it should be’, some women did not understand what this question was trying to ask:

*“I suppose I think straight away as in “that was the way I wanted it to go”. But yeah that is what would spring to mind. That all had gone as I wanted it to go or my birth plan had gone completely out the window.” Q28*

However, for those that did, they related it either to the labour and birth going the way they had planned or the ‘rite of passage’ of a woman to give birth:

*“I can see how it fits with the way I interpreted natural in terms of the whole sort of rites of passage for womanhood, but I would struggle with that and the wording with that question a bit more, I don’t know how to interpret it.” Q30*

(7) ‘Fantasies my child will die during labour’, although everyone understood what this question was trying to ask, for many the word ‘fantasies’ relates to something positive happening and therefore all women felt that this word did not make sense in this context.

*“Yeah, I would say that’s a fair question. I probably say more thoughts. Fantasies kind of suggests something that you … now that I reread it, fantasies is like what I want to happen” Q10*

Therefore, overall 10 items of the 33 items were identified as having limitations in women’s understanding.

#### OWLS

In the Oxford Worries about Labour Scale (OWLS), only 1 of the 10 items created concerns about understanding and this was only identified by women in the high group (1) ‘Not knowing how long labour would take’, all of the women in the low group framed this item as more of a curiosity rather than a fear, but for some women in the high group the item was associated with the fear of a long labour rather than the not knowing element indicated in the question.

*“No not fear, curiosity but not fear”*

#### SPECS

In the Slade-Pais Expectations of Childbirth Scale (SPECS) of the 10 items only one item was ambiguous for women to understand (1) ‘Labour is unknown’, many women did understand this item, however a couple of women found it more difficult to clarify what it was asking:

*“Don’t really understand what that means. Like the unknown … like you don’t know. I’m not sure I would know how to answer that? I would take that out, that doesn’t really say anything to me.”*

#### FOBS

In terms of the FOBS, no items were difficult to understand. All women found the words ‘calm’, ‘worried’ and ‘fear’ easy to understand.

Understanding in terms of reading ease:

The FOBS had the highest Flesch reading ease level (81.9%), followed by the OWLS (69.1%), and then the WDEQ-A and SPECS both at (66%).

### Acceptability of measures

Although, overall, most women viewed all four measures positively, there were limitations with each that challenge their suitability for general use.

#### Phraseology

In terms of acceptability in phraseology many liked the language/phraseology in the OWLS and felt that it was more colloquial so they could identify with it easily. There were 2 items in the WDEQ-A and 1 item in the SPECS that raised acceptability issues for women, although women were clear that their concerns related to the strength of negativity associated. ‘Dangerous’ and ‘Deserted’ from the WDEQ-A, and ‘My body will fail me during labour’ from the SPECS were perceived as overly negative phraseology for women and therefore less acceptable in a measure of FOC as women felt they might increase fear.

*[‘Dangerous’, WDEQ] “It’s a bit of a strong word I think. I suppose I would go back … not dangerous I wouldn’t, but safe maybe. Safe is better maybe … Because if you say dangerous you’d panic wouldn’t you? That’s a panic word for me really you know? Whereas safe is a little bit more ‘oh okay then’ but if someone says that that’s dangerous then you are thinking ‘oh what’s happening?’ That sort of panic kicks in doesn’t it? So, no I would definitely say safe and not dangerous.” Q2*

*[‘Deserted’, WDEQ] “You’re going to be in a hospital, in a strange room and then all of a sudden everyone is going to leave you on your own. Deserted is a little bit scary … No, no that’s not really come into my mind at all. I wouldn’t have that, it’s a bit scary.” Q3*

*[‘My body will fail me during labour’, SPECS] “Ooh I don’t like that! It’s this one it’s the way it is worded. I understand because it is the expectation you have of yourself. And you could easily be frightened of not having the confidence in yourself to achieve what you expect from labour if that makes sense? But it’s just really dark to go into that and try to quantify that. It’s really full on.” Q22*

Further concerns around phraseology in the SPECS included the item ‘I will not be able to give birth naturally’ as most of the women reported that to ‘give birth naturally’ would mean a vaginal delivery. They understood the question and could score it; however, several women from the low group felt that the wording made the suggestion that having a cesarean section is ‘unnatural’ which could potentially upset some women.

Also, some suggested that the phraseology of the items should focus on how a pregnant woman is currently feeling about labour and childbirth rather than how they imagine they will feel in the future, as many found this too complex to rate.

#### Presentation and ease of use of each measure

Many felt that the practicality of the OWLS questionnaire made it easier and quicker to answer than the WDEQ-A. The women also liked the layout, as it was less ‘busy’ with a clear structure. Some women liked the visual analogue of the FOBS however most women preferred the 5-point Likert scale rather than a 4 point scale of the W-DEQ because it allowed them the option to score in the middle. Some women suggested that a Visual Analogue Scale might be a good opening to the measure (i.e., to be placed at the start of a longer assessment tool), to ease people into the process or initiate further discussion.

#### The breadth of items included in each measure

One of the main reported positive aspects of the WDEQ-A was a perception that it was a thorough tool which is good to elicit what people are feeling pre-delivery and captures most of women’s fears. However, overall the OWLS was the favoured questionnaire by the majority of the women due to its perceived relevance. Although some liked the thoroughness of the WDEQ-A, others felt it was too long and repetitive and some women felt it was too heavily weighted towards the emotional rather than physical side of labour. In contrast to the WDEQ-A, some women felt that the OWLS was too weighted towards the worries about physical aspects of labour and childbirth rather than the emotional components. Some also felt that the OWLS and SPECS failed to ask questions about the woman’s safety and generally were not sufficiently detailed and therefore might not capture all of the fears that women might have around childbirth. Similarly, with the FOBS many women in the high group identified with the word fear and felt that they could place themselves high on the fear scale, however the women in the low group felt that the scale did not give enough detail about what the fears are for women and therefore found it less useful.

*“But that seems really basic and just having the two words, not everyone’s intermediate for calm and worried and no fear and fear is the same. If I placed a line in the middle and someone else did it, it doesn’t mean we think the same thing about what that represents? And I wouldn’t know where to put that mark”*

Some suggested that the lack of detail does not allow professionals to understand what support the woman needs nor does it challenge the women to think more deeply about their fears. Interestingly, for some women with high fear they felt that this might communicate to women that professionals are not taking their anxiety seriously.

This suggests that a measure needs to include a mixture of potential emotional worries, and physical concerns, to maximise its acceptability as a measure of FOC.

### Content validity of each measure

Content validity refers to the extent to which items within a measurement tool adequately reflect each domain of a construct [36]. Following the previous stages, all questionnaires were reviewed against the 10 domains identified in Slade et al. [13] to assess content validity. Table 2 shows the mapping process and emissions within the 4 measures assess against the 10 domains for FOC. All items of each scale were systematically reviewed against the each of the 10 elements by the research team experienced in the field: a consultant midwife (GH) two clinical psychologists (PS KB) and a research psychologist (KS). This continued until a consensus on each item was reached.

**Table 2:** Comparison of item content within each scale with the 10 key elements of FOC as identified by Slade et al. [13]

FOC Element	WDEQ-A	OWLS	SPECS	FOBS
1. Fear of not knowing and not being able to plan for the unpredictable	Totally as it should be	Getting to the hospital in time Having a long labour Not knowing how long labour will take Not knowing when I would go into labour	Labour will be complicated	
2. Fear of harm or stress to the baby	Fantasies child will die Fantasies child will be injured		I am worried about the health of my baby	
3. Fear of inability to cope with pain	Pain	Getting effective pain relief Pain and discomfort of labour		
4. Fear of harm to self in labour and post-natally	Safe Dangerous			
5. Fear of being ‘done to’		Needing a caesarean Having ventouse or forceps delivery Having to be induced	Labour will be complicated I worry I will need emergency surgery	
6. Fear of not having a voice in decision making				
7. Fear of being abandoned/alone	Lonely Deserted Abandoned Trust			
8. Body’s ability to give birth	I will allow my body to take control Natural		My body will fail me during labour I will not be able to birth naturally	
9. Losing control	Composed Panic I will behave badly I will totally lose control of myself	Embarrassment		
10. Generic fear of unknown			Labour is unknown	Fear-no fear

The WDEQ-A mapped onto 7 of the 10 domains but did not include (1) Fear of being ‘done to’, (2) Fear of not having a voice in decision making and (3) Generic fear of unknown.

The OWLS mapped on to 4 domains but did not include (1) Fear of harm or stress to the baby, (2) Fear of harm to self in labour and post-natally, (3) Fear of not having a voice in decision making (4) Fear of being abandoned/alone, (5) Body’s ability to give birth and (6) Generic fear of unknown.

The SPECS fear subscale mapped on to 5 domains but did not include (1) Fear of inability to cope with pain, (2) Fear of harm to self in labour and post-natally, (3) Fear of not having a voice in decision making (4) Fear of being abandoned/alone and (5) Losing control. As the full version of the SPECS questionnaire (not just the fear subscale) was also available this was checked to review whether the missing domains were present. If the full scale were to show appropriate content validity then future research could further review its clarity and acceptability. Although there were items in the full SPECS that did reflect all 10 domains, items for two of the domains were felt to demonstrate inadequate specificity. The domain “Fear of harm or stress to the baby” was tentatively covered in the SPECS with the item “I will be worried about the health of my baby” but the team agreed that this did not cover fears about injury to the baby or death, which were clearly outlined in the FOC domains. In addition ‘Fear of harm to self in labour and post-natally’ was covered in the SPECS by two items “I worry about trauma to my body” and “My body will hurt during labour” The team consensus was this might not reflect the level of concern about post-natal ongoing injuries e.g., fear of impact on sexual relationships and incontinence etc. The FOBS tentatively mapped onto one domain (1) Generic fear of unknown, however due to its brevity it did not generally map onto the other domains.

Good content validity requires a measure to address all relevant domains of a construct [36]. None of the four main existing measures will ask women about every element of the FOC construct and therefore is indicative of limited content validity for measurement of FOC in this small sample of UK women.

## Discussion

In summary, no widely utilised measure of FOC fulfilled requirements for adequate clarity of understanding and acceptability and (following initial examination) content validity for the measurement of fear of childbirth in a UK population. Women valued tools that were worded in a way that was easy to understand, and where items focused on their current feelings rather than their expectations of giving birth. Although women felt that scoring measures using a visual analogue scale (as with the FOBS) was useful, there was a preference for tools that ask about both physical and emotional concerns for birth using a 5 point likert scale to enable a neutral response where appropriate.

Acceptability and understandability of items within a measure could potentially be improved via alterations to the wording of certain items (with the permission of authors). However many of the currently used scales are missing crucial components for valid measurement of FOC within the UK population. It is clear that effective tools not only need to consider the content but also the structure and accessibility of the language for women [6, 7]. In addition to issue of content validity this study substantiates previous studies that suggest that clarity and understandability can be compromised when measures are translated directly from other languages, and this needs to be carefully considered before such measures are routinely adopted at service level.

Acceptability of administration also needs consideration and women wanted adequate coverage without undue length and without all items being framed in a highly negative way. Whilst the FOBS may be acceptable as a first screening tool it was not felt to show and indeed did not incorporate sufficiently detailed coverage to be useful alone. It is also notable that many of the women involved (in both the high and the low fear group) felt strongly that women should be routinely asked about their fears around childbirth, which is consistent with the literature [38]. In summary this work suggests that a new measure of FOC would be beneficial for use with UK women.

### Limitations

The study included a small group of women to review the measures, however the sample size is reflective of recommendations for the cognitive interview technique [32, 33] and consistent alignment in perspectives from both groups highlight that the current measures do not assess all elements of FOC that are identified within UK samples [13, 38]. It is acknowledged that the findings derive from a small UK sample and in keeping with the methodological approach saturation was not a requirement [32]. This is in line with Saunders et al. [39] recommendation that saturation should only be included where its purpose is conceptually and theoretically supported. Presence of FOC was inferred via self-report, and therefore categorisation into high and low fear was led by participants’ own perspectives about giving birth. Given the uncertainty in available cut offs on scales for UK populations and the aims of the present study, quantitative assessment of fears to inform recruitment was not used. Several participants were recruited via hospital social media and therefore confirmation of high fear by clinical judgement of the specialist midwives was not available. Extending to this method of recruitment was advised by our service user organisation because of their awareness of women’s difficulty in disclosure of fear to health professionals. However for all women high fear and low fear was confirmed by the clinical judgment of an experienced clinical psychologist (KB). The average age of participants was slightly higher than the UK average (29 years [40]),

The measures considered were all considered in the same order; this provided a standardisation of process but could also potentially lead to carryover effects. To facilitate participation, a pragmatic approach to interview location was adopted based on the participants’ preferences but it is recognised that variations in interview locations could introduce confounding influences. Participants were in their second or third trimester; further examination or development of measurement tools to identify FOC should examine the timing of measurement during pregnancy. This study does not address other forms of validity such as predictive, discriminant or construct validity. It should be noted that the limitations identified in existing tools related specifically to a small sample of UK women, and findings may differ with women in other countries.

## Conclusion

Findings from the current study highlight limitations in the content validity, comprehension and utility of existing measurement tools to identify FOC in UK women. There is a need either for extensive adaptation of existing measures or the development of a specific fear of childbirth tool which is clear, demonstrates appropriate content validity in covering the 10 dimensions identified, and is acceptable (in terms of presentation and length). This will allow accurate identification of women who are fearful of childbirth will allow healthcare professionals to activate an early and effective pathway of care.

## Data Availability

The datasets used during the current study are available from the corresponding author on reasonable request.

## Abbreviations

FOBS: Fear of Birth Scale
FOC: Fear of Childbirth
OWLS: Oxford Worries about Labour Scale
SPECS: Slade-Pais Expectation of Childbirth Scale
WDEQ-A: Wijma Delivery Expectancy/ Experience Questionnaire Version A
